# Hospitals and State Aid

**Published:** 1911-06-17

**Authors:** 


### HOSPITALS AND STATE INSURANCE.

### HOSPITALS AND STATE AID.

### CONDITIONS UNDER WHICH GOVERNMENT AID IS GIVEN TO HOSPITALS.

(BY OUR SPECIAL COMMISSIONER.)

IN response to an inquiry with reference to the conditions under which hospitals in the oversea Dominions and elsewhere enjoy certain grants in aid from the State, we have compiled the following summary, which we trust will be of use to hospital workers at the present moment. The precis has been made, wherever possible, from official sources, and may be relied upon as accurate, although it must be borne in mind that the figures available are for 1908 only. It is our intention to publish next week a further instalment showing the conditions which obtain in New Zealand, Canada, South Africa, the Crown Colonies, and India. We are of opinion that everything which is likely to be of practical value to hospital workers at the present crisis should be published, and we appeal to readers to make this series as complete and detailed as possible. Any information on the subject dealt with in these articles will be gratefully acknowledged by our Special Commissioner. Letters should be clearly marked "Hospitals and State Aid" on the envelope, so that they can receive prompt attention in order that the information may be incorporated in the succeeding articles of the series.—ED. THE HOSPITAL.

### THE COMMONWEALTH OF AUSTRALIA.

The hospital system in Australia is in a large measure modelled on that which exists in this country. There are, however, interesting features which are worth special study. For example, the laws relating to public health and the administration of charity are much more complete and much better consolidated than our own legislative enactments which deal with similar subjects. The Minister of Public Health is a reality, not a fond imagining, in Australia, and the good that his department has done in the past can easily be estimated by anyone who takes the trouble to glance through the exceedingly instructive reports which emanate from the different Government offices which are under his control. Hospital revenue in the Commonwealth is derived from various sources : from voluntary contributions similar to those in this country, including grants from Hospital Sunday and Saturday Funds, from charity organisation societies, from special and religious bodies, from private donations and bequests, and from special entertainments and social functions organised for the benefit of the charities; from payments by private patients admitted to private wards, or from ordinary receipts from in-patients, and to a smaller extent from out-patients' contributions; from endowments and interest accruing from investments; and finally from grants made by Government according to a fixed scale in the majority of cases, and from similar grants made by municipalities or corporations.

The Official Year-Book of the Commonwealth of Australia,[1] containing authoritative statistics for the period 1901-1909, and published under the authority of the Minister of Home Affairs, gives the fullest and most complete summary of the conditions under which the hospitals of the Commonwealth, considered as a whole, exist at present. Section XXIV., dealing with Public Benevolence, deals fully with these conditions, and the following excerpts are taken from it :—

"Charity and charitable effort in Australia may be classified under three headings, viz. (*a*) State; (*b*) public; and (*c*) private. To the first belong all institutions wholly provided for by the State; examples are the lunatic asylums of the various States, the various Government hospitals in Western Australia, and the Government asylums for the infirm in New South Wales. The second class include public institutions of two kinds, viz. : (1) Institutions partially subsidised by the State or State endowed, but receiving also private aid; and (2) those wholly dependent upon private aid. To the former division belong such institutions as the Melbourne and other large metropolitan hospitals. Of the latter example are institutions established and endowed by individuals for the benefit of the needy generally. To the third class belong all charitable movements of a private or special character. A more or less accurate statistical account is possible in classes 1 and 2, but with regard to 3 it may be said that, for obvious reasons, no tabulation is possible. Public response to special appeals and summary relief in kind, often considerable, is nevertheless not statistically recorded. Hospitals, orphanages, homes, benevolent asylums, etc., form, of course, the main channels in which the current of charity flows. There are nevertheless numerous other and minor charities, perhaps less definitely established and less frequently noticed, which mark the course and measure the amount of a considerable volume of private beneficence. In institutions which receive Government aid, management and finance are usually relegated to executive bodies ordinarily elected on a democratic basis."

#### SOCIAL CONDITIONS IN AUSTRALIA.

"The distribution of wealth in the Australian Commonwealth, and the generally favourable condition of Australia as regards scope for the exercise of natural ability, operate to prevent the development of a permanent pauper class, and at the same time lessen in a dual way the burden of charity. This latter is brought about by the increase, on the one hand, of the number of people whose prosperity enables them to relieve the indigent and unfortunate, and by the reduction, on the other, of the number who need assistance. Enactments of State Legislature have decreed short hours and a liberal holiday allowance for large numbers of persons engaged in industrial and other pursuits, and, even in occupations not covered by Act of Parliament, the general conditions of employment often provide a considerable amount of leisure. This, and an equable climate, enable the community to spend much of its time in the open air, thus reducing the ravages of disease and the scourge of epidemic. No poor rate is levied in Australia, and Government aid, without return, is required only for the aged and disabled. The only States which have had an old age pension system are New South Wales, Victoria, and Queensland; but in June 1908 legislation by the Commonwealth Parliament (Invalid and Old Age Pensions Act, No. 17 of 1908) provided for the payment of old age pensions throughout the Commonwealth as from July 1, 1909, or such earlier date as may be enacted, and the Old Age Pensions Appropriation Act (No. 18 of 1908) appropriated £750,000 for invalid and old age pensions."[2]

#### CHARITY REFORMS.

"Lately the evident overlapping of charitable effort," remarks Mr. Knibbs, in his interesting chapter, "has led to discussion regarding the methods of collection and distribution. The proportion absorbed in expenses, alleged to be unduly large, has also given rise to a desire for improved administration. An important conference of representatives of the charitable associations was held in Melbourne in September 1907. It was the initiation of an effort to digest systematically the experiences of the committees of management of the various hospitals and kindred institutions. These obtain their revenue from State and municipal subsidies, from proceeds of concerts, entertainments, etc.; from organised public collection, from private contributions and bequests and from patients. Collectors are in some cases paid, in others not. Frequently institutions similar in character enter into competition for subscriptions. The result is overlapping both in organisation and expenditure. It has been officially stated that far too small a proportion of the money which the generosity of subscribers furnishes benefits the real sufferers. The public eleemosynary impulse is probably also prejudiced by the utilisation of institutions for the poor or destitute by classes who can afford to pay for medical or surgical treatment, as in the case, for example, of hospitals. Organisation and co-ordination would make available for the sick and needy large sums which are now spent in the upkeep of redundant buildings and the maintenance of unnecessary officers and servants. Societies have accordingly been formed to prevent overlapping. Other proposed reforms take into account the origins of poverty and crime, seeking to remedy the underlying causes."

Dealing more specially with the hospitals of the Commonwealth, the same authority remarks: "Most of the State capitals have several large and well-equipped hospitals, and there is at least one (hospital) in every important town. In large centres there are hospitals for 'specials'—consumptives, women, children, infectious diseases, incurables, etc."

The following tabulation gives the statistics so far as the hospitals are concerned. In the tables for 1904, 1905, and 1906 hospitals for "specials" are included, but the tables for 1907 and 1908 give only the general hospitals :—

*Australian Hospitals* 1904 *to* 1908.

| | 1904 | 1905 | 1906 | 1907 | 1908 |
|---|---|---|---|---|---|
| Number of Hospitals ... | 304 | 308 | 313 | 304 | 312 |
| Number of Beds .. | 71,384 | 11,778 | 12,108 | 11,463 | 12,057 |
| In-patients ... ... | 96,748 | 101,200 | 106,488 | 104,483 | 114,666 |

The following table, also taken from Mr. Knibbs' book, gives the State expenditure on all charities (inclusive of hospitals, mental hospitals, asylums, and other charities, both State and public). The total expenditure in the Commonwealth on such charities, including funds raised from all sources, but exclusive of old age pensions, is estimated at £17,000,000 for the year 1908 :—

*General Revenue of Australian Hospitals*, 1908.

| | Commonwealth. | New South Wales. | Victoria. | Queensland. | South Australia. | West Australia. | Tasmania |
|---|---|---|---|---|---|---|---|
| Patients' Fees, Etc. ... | £80,977 | 38,877 | 15,705 | 14,111 | 3,260 | 9,024 | |
| Other Income from all sources except Government contributions ... ... | 274,936 | 99,567 | 83,763 | 59,592 | 9,227 | 11,241 | 11.546* |
| Govt. Grants ... | 397,328 | 132,882 | 53,097 | 71,847 | 38,777 | 86,130 | 14,595 |

(*Includes patients' fees.)

The six States which comprise the Commonwealth are New South Wales, Victoria, Queensland, South Australia (Northern Territory), Western Australia, and Tasmania. The conditions obtaining in each State with regard to hospitals and the subsidies they receive from the State vary considerably, so that it is necessary to deal with each division in some detail.

### VICTORIA.[3]

The total number of organisations throughout the State which administered charitable relief, or were of a reformatory character, and which forwarded returns to the Government statist for the year 1909, was 248, of which 179 received Government aid. The total receipts were £965,400, of which Government contributed £695,775, while £269,625 was received from all other sources. The hospitals alone, seventy in number, received a total of £418,460, of which sum the Government contributed £252,209, the receipts from all other sources, including endowments, totalling £166,251. The average cost for each inmate in the general hospitals works out at £60 19s. 9d., in this calculation the cost of out-patient treatment being necessarily included, as there is no available information showing the cost of in-patients and out-patients separately. The Government contribution to a hospital is generally made on the pound for pound principle, but special grants are made in certain cases. The Year-Book contains an interesting summary of the chief hospitals, detailing their history and giving full accounts of their finances. In addition the hospitals receive grants from the municipalities of the various towns, such grants being generally calculated according to the needs of the institutions; special grants are made in cases where improvements, additional buildings, etc., are deemed necessary. The State exercises a general supervision over the hospitals receiving State assistance, but does not interfere in the management or administration.

### NEW SOUTH WALES.[4]

Somewhat similar conditions obtain in this State. At the close of the year 1908 there were 137 hospitals in operation in the State, which, most of them, received large grants from Government. The Government Medical Officer may assign cases, under Government orders, to the hospitals; during the year 1908, 9,673 such orders were granted out of a total of 47,349 patients treated in the hospitals. The total revenue of all hospitals, with the single exception of that at Little Bay, during the same year was £253,139, of which £112,860 was received from Government. The expectation that the introduction of old age pensions would cause a reduction in the expenditure of the Government on charity has not been realised. The interesting tables given in Mr. Trivett's book ought to be consulted by everyone who studies this subject, since the conclusions have a practical value for us at this time. The Invalidity Pensions Act of this State, passed in 1907, allows the grant of a pension not exceeding £26 per annum to persons permanently disabled by sickness or accident provided such persons are not inmates of a charitable institution. This Act has been abrogated by the passing of the Commonwealth Act in 1908, under which the invalidity clauses have been postponed until such time as they are specially enforced by proclamation. The section dealing with the work of the hospitals in relation to the friendly societies and their sick pay is also interesting and well worth study. In New South Wales, the Government grants to hospitals are on the pound for pound principle, calculated on voluntary contributions, exclusive of patients' payments and receipts from endowment.

### WESTERN AUSTRALIA.

The figures for this State have already been given. (See table.) There are twenty-six Government hospitals wholly supported out of the State revenue, and fourteen general hospitals which are public institutions, governed by elective boards, and receiving State assistance in the shape of grants. The system under which such grants are made is as follows: The State gives a pound for pound subsidy on all donations, subscriptions, and net proceeds of entertainments; it pays 25s. per week per patient for indigent patients, and gives further a grant of £100 a year towards the salary of every medical officer at all hospitals with the exception of the hospital at Nannine, where the grant is £150. The large public hospitals at Perth and Fremantle are managed by special elective committees and do not fall under this system, since by special Act of Parliament they are managed on a different system and are mainly supported by the State.[5]

### SOUTH AUSTRALIA.

The figures concerning the revenue of the hospitals in this State have already been given. The Government grants are calculated partly on a modified pound for pound principle and partly on the amount of money required for special building purposes. As a matter of fact the contribution of the State is almost double the amount raised by voluntary effort. As in the other States the Government has the right to inspect the hospitals and to suggest emendations with regard to new buildings, etc. It does not interfere in the management of the public hospitals, which are entirely controlled by elective bodies, chosen by the subscribers.

### QUEENSLAND.

There are at present over seventy subsidised hospitals which receive yearly grants. These vary in size and importance from hospitals like the Brisbane General Hospital with 250 beds to small local or cottage hospitals. They are managed by committees elected locally by the subscribers in nearly all cases. An exception is the hospital already mentioned, where the Government has reserved and exercised the right of nominating four out of the seven members of the Board of Management. Subscriptions collected locally are subsidised by the State on the £2 for £1 principle, but from this contribution by Government special subscriptions for new buildings are excluded, such special subscriptions being subsidised at the rate of pound for pound only. "The State subsidy," remarks Dr. Hare, the Inspector-General of Hospitals, in his interesting article in the official Year-Book, "on voluntary charitable contributions was instituted to preserve the charitable principle of hospital relief in a new country where the unequal territorial distribution of wealth renders it impossible for many of the smaller communities to provide themselves with hospitals." Subscriptions to benevolent and charitable associations and societies are subsidised at the rate of £2 for every £1 raised, up to the first £75 raised; after that figure has been reached the State augments the amount subscribed by an equal sum on the pound for pound principle. Hospitals are inspected by the State Inspector, but there is no further Government interference.

### TASMANIA.

General hospitals are subsidised by the State on what is practically the pound for pound principle. The exceptions are the hospital for contagious diseases, which is wholly supported by the Government and managed by State officials. Public hospitals are governed by elective bodies, but are subject to Government inspection, and the grant may be withdrawn if the inspector's report is adverse.

[1] Official Year Book of the Commonwealth of Australia, No. 3, 1910. Published under the authority of the Minister of Home Affairs. By G. H. Knibbs, F.R.S.S., etc., Commonwealth Statistician. (McCarron, Bird and Co., printers, Melbourne.) C.S. No. 85.

[2] The Invalidity Clauses of the Invalid and Old Age Pensions Act of 1908 have not yet come into operation; the date when they will come into force is to be fixed by special proclamation.

[3] Victorian Year Book 1909-10. By A. M. Laughton, F.I.A., F.F.A., F.S.S., Government Statist. Published by authority. J. Kemp, printer, Melbourne.

[4] The Official Year Book of New South Wales, 1908-09. By John Trivett, F.R.A.S., F.S.S. Published by authority. 1910.

[5] See reports of Superintendents of Charities and Inspector of Industrial and Reformatory Schools; also Official Year Book, Reports of Public Hospitals, and Annual Report of Department of Public Health.

